# Salt Stress Tolerance of Dark Septate Endophytes Is Independent of Melanin Accumulation

**DOI:** 10.3389/fmicb.2020.562931

**Published:** 2020-12-10

**Authors:** Dalia A. Gaber, Charlotte Berthelot, Iris Camehl, Gábor M. Kovács, Damien Blaudez, Philipp Franken

**Affiliations:** ^1^Leibniz-Institute of Vegetable and Ornamental Crops, Grossbeeren, Germany; ^2^Institute of Biology, Humboldt University, Berlin, Germany; ^3^Université de Lorraine, CNRS, LIEC, Nancy, France; ^4^Department of Plant Anatomy, Institute of Biology, Eötvös Loránd University, Budapest, Hungary; ^5^Plant Protection Institute, Centre for Agricultural Research, Budapest, Hungary

**Keywords:** *Periconia macrospinosa*, *Cadophora* sp., *Leptodontidium* sp., albino mutants, tricyclazole, scytalone dehydratase-encoding gene

## Abstract

Dark septate endophytes (DSEs) represent a diverse group of root-endophytic fungi that have been isolated from plant roots in many different natural and anthropogenic ecosystems. Melanin is widespread in eukaryotic organisms and possesses various functions such as protecting human skin from UV radiation, affecting the virulence of pathogens, and playing a role in development and physiology of insects. Melanin is a distinctive feature of the cell walls of DSEs and has been thought to protect these fungi from abiotic stress. Melanin in DSEs is assumed to be synthesized via the 1,8-dihydroxynaphthalene (DHN) pathway. Its function in alleviation of salt stress is not yet known. The aims of this study were: (i) investigating the growth responses of three DSEs (*Periconia macrospinosa*, *Cadophora* sp., and *Leptodontidium* sp.) to salt stress, (ii) analyzing melanin production under salt stress and, (iii) testing the role of melanin in salt stress tolerance of DSEs. The study shows that the three DSE species can tolerate high salt concentrations. Melanin content increased in the hyphae of all DSEs at 100 mM salt, but decreased at 500 mM. This was not reflected in the RNA accumulation of the gene encoding scytalone dehydratase which is involved in melanin biosynthesis. The application of tricyclazole, a DHN-melanin biosynthesis inhibitor, did not affect either salt stress tolerance or the accumulation of sodium in the hyphae. In addition, melanin biosynthesis mutants of *Leptodontidium* sp. did not show decreased growth performance compared to the wild-type, especially not at high salt concentrations. This indicates that DSEs can live under salt stress and withstand these conditions regardless of melanin accumulation.

## Introduction

In natural ecosystems, all higher plants can be colonized by endophytic organisms mostly by fungi and bacteria (Li et al., 2008). Dark septate endophytes (DSEs) are ubiquitously occurring root-colonizing fungi characterized by melanized, septate hyphae (Jumpponen, 2001) belonging to different orders of the phylum Ascomycota. DSEs comprise conidial and sterile fungi (Jumpponen and Trappe, 1998). They were detected in different arid, temperate, arctic, tropical, boreal, or alpine ecosystems often characterized by abiotic stress conditions (Jumpponen and Trappe, 1998; Mandyam and Jumpponen, 2005; Rodriguez et al., 2009), but have been also detected in managed soils in the absence of abiotic stress (Andrade-Linares et al., 2011). Around 600 plant species from 320 genera and 114 families were reported to be colonized by DSEs (Jumpponen and Trappe, 1998; Mandyam and Jumpponen, 2005). The complete genomes of several DSEs like *Microdochium bolleyi* (David et al., 2016), *Harpophora oryzae* (Xu et al., 2014), and *Phialocephala subalpina* (Schlegel et al., 2016) are available and comparative genome analyses has been also published for *Periconia macrospinosa* and *Cadophora* sp. (Knapp et al., 2018). These genomic data are important contributions to a better understanding of the biology of DSEs including the mechanisms of abiotic stress tolerance and their interaction with plants.

Abiotic stresses negatively affect the survival and productivity of crops. Ectomycorrhizal fungi can protect plants against harsh environments with their hyphal mantle surrounding the roots, but also many other root-colonizing fungi [e.g., arbuscular mycorrhizal fungi (AMF), representatives of the order Sebacinales and DSEs] are known to confer abiotic stress tolerance to a wide range of plants (Ruiz-Lozano et al., 1996; Mandyam and Jumpponen, 2005; Waller et al., 2005; Sirrenberg et al., 2007; Baltruschat et al., 2008; Porras-Soriano et al., 2009; Hajiboland et al., 2010; Newsham, 2011; Bitterlich et al., 2018). Accordingly, stress tolerance of root endophytes themselves is a prerequisite for a successful symbiotic relationship with plants under such conditions to improve their tolerance to abiotic stress. This suggests that root-colonizing fungi adapted to harsh environments might be better in conferring abiotic stress tolerance than related strains evolved at non-stressed conditions. DSEs received much attention because they are supposed to have an important role in the alteration of host water uptake and to be involved in host drought, salinity, and heat tolerance (Jumpponen, 2001; Mandyam and Jumpponen, 2005). DSEs could be therefore especially interesting for application as inocula in cropping systems under abiotic stress. As these fungi have been described to have also no or even negative effects on plant performances (Mayerhofer et al., 2013), it will be important to select DSEs which can be used for application.

Tolerance against non-physiological salt concentrations in the environment is generally based on two types of mechanisms (Ruppel et al., 2013). The avoiding strategy includes on the one hand specific cell wall constructions in order to prevent influx of salt ions and water loss from the cells. On the other hand, particular ion pumps facilitate ion extrusion back into the environment. The intracellular adaptation strategy is mainly based on the accumulation of compatible solutes, but also adaptations of particular proteins and enzymes are involved.

It is discussed that melanin plays an important role in heavily pigmented fungi to protect plants against detrimental effects of environmental stressors like drought, heat, high concentrations of salts, heavy metals, and radiation (Gessler et al., 2014). Concerning salt stress, Qin et al. (2017) isolated 27 representatives of seven families of pleosporalean DSEs from halophytic plants growing in Chinese saline areas. Subsequently, they investigated their responses to environmental stresses like pH, temperature, ionic stress induced by adding ionic osmolytes such as NaCl and KCl and osmotic stresses by applying sorbitol. Those and other authors found that DSEs show a wide pH and salt tolerance, but also particular sensitivities depending on the species (Postma et al., 2007; Qin et al., 2017; Santos et al., 2017). Fernandez and Koide (2013) showed that melanin biosynthesis inhibition had no impact on growth rate of three isolates of the strongly melanized ectomycorrhizal fungus *Cenococcum geophilum* under normal conditions. The growth rates were, however, negatively affected at an osmotic potential of −1.7 MPa.

Three pathways of melanin biosynthesis have been detected in fungi (Gessler et al., 2014; Supplementary Figure 1) and it has been recently shown for three different DSE species that melanin is mainly produced through 1,8-dihydroxynaphthalene (DHN) (Berthelot et al., 2020). In this DHN pathway, 1,3,6,8-tetrahydroxynaphthalene (THN) is synthesized by the action of a polyketide synthase from acetyl CoA or malonyl CoA as precursors. Then, the intermediates scytalone, 1,3,8-THN and vermelone are formed through reduction and hydration reaction series (Bell and Wheeler, 1986; Wheeler and Klich, 1995). A key enzyme involved in these reactions is the scytalone dehydratase which catalyzes the dehydration of scytalone to 1,3,8-THN. Finally 1,8-dihydroxynaphthalene is produced and is converted to melanin by polymerization (Butler and Day, 1998; Langfelder et al., 2003). Tricyclazole, known for over 30 years as fungicide, is a specific inhibitor of this melanin biosynthesis pathway because it inhibits the formation of the intermediate compound vermelone (Wheeler and Klich, 1995).

The presence of melanin in cell walls of fungal hyphae growing in harsh environments is a common phenomenon (Butler and Day, 1998; Robinson, 2001). Melanin is localized either external to or within the cell wall as it was shown by examination of hyphal walls ultrathin sections of four fungal species (*Amorphotheca resinae, Epicoccum nigrum, Humicola grisea*, and *Colletotrichum coccodes*) (Ellis and Griffiths, 1974). In other fungi like *Cryptococcus neoformans*, melanin is deposited throughout the cell wall to form concentric rings (Mandal et al., 2007). In some fungi, melanin is localized in the inner layer of the cell wall and associated to the plasma membrane, like in the appressorium of the rice blast-causing fungus *Magnaporthe oryzae* (Fernandez and Orth, 2018). Based on the localization, melanin could be involved in the exclusion of toxic compounds including sodium ions and being therefore part of the machinery involved in salt stress tolerance.

The objective of the present study was the investigation of the role of melanin in the tolerance of DSEs to a moderate salt stress. *P. macrospinosa*, *Cadophora* sp., and *Leptodontidium* sp. were selected, because they belong to different taxonomic groups and were isolated from two different biomes with moderate salt concentrations. Additional reasons for the selection were the availability of genome sequences of *P. macrospinosa* and *Cadophora* sp. and of the albino mutants of *Leptodontidium* sp. First, the responses of these three DSEs to increasing salt concentrations were examined. Based on this, the hypothesis was tested that melanin had a role in salt stress tolerance of DSEs (i) by analyzing if melanin production is induced by salt and, (ii) by investigating the impact of a specific melanin biosynthesis inhibitor on salt stress responses, and (iii) by the application of albino mutants of *Leptodontidium* sp., which do not synthesize melanin.

## Materials and Methods

### DSEs Used in This Study

Three strains of DSEs were used in the current study (Table 1). *P. macrospinosa* (DSE 2036) and *Cadophora* sp. (DSE 1049) were isolated from semi-arid grasslands of the Great Hungarian Plain (Knapp et al., 2012, 2015). *P. macrospinosa* is a well-known pleosporalean DSE belonging to *Periconiaceae*, Pleosporales (Tanaka et al., 2015). *Cadophora* sp. (DSE 1049) is a helotialean root endophyte that probably represents the recently described species *Cadophora meredithiae* (Walsh et al., 2018). *Leptodontidium* sp. (Me07), was isolated from root samples of poplar trees which were collected from a Zn- and Pb-polluted soil located in the north of France (Berthelot et al., 2016). This strain clustered with sequences of the previously described root endophytes *Leptodontidium orchidicola* and the reference *Leptodontidium* sp. PMI 412 strain (Berthelot et al., 2016). We also involved five isogenic albino mutants of *Leptodontidium* sp. in the study: Δ1110, Δ1113, Δ434, Δ1145, and Δ521 (Table 1). These mutants were derived from the Me07 strain by T-DNA insertion mutagenesis followed by phenotypical screening (Berthelot et al., 2017). The three fungi form dark septate hyphae and microsclerotia when colonizing roots (Knapp et al., 2012; Berthelot et al., 2016).

**TABLE 1:** List of dark septate endophytic fungal strains used in this study.

Species	Strain	Order	References
*Periconia macrospinosa*	DSE 2036	Pleosporales	Knapp et al., 2015
*Cadophora* sp.	DSE 1049	Helotiales	Knapp et al., 2012
*Leptodontidium* sp.	Me07	Helotiales	Berthelot et al., 2016
*Leptodontidium* sp. (Mutant)	Δ1110	Helotiales	Berthelot et al., 2017
*Leptodontidium* sp. (Mutant)	Δ1113	Helotiales	Berthelot et al., 2017
*Leptodontidium* sp. (Mutant)	Δ434	Helotiales	Berthelot et al., 2017
*Leptodontidium* sp. (Mutant)	Δ1145	Helotiales	Berthelot et al., 2017
*Leptodontidium* sp. (Mutant)	Δ521	Helotiales	Berthelot et al., 2017

### Growth of DSEs Under Salt Stress

Preliminary experiments were performed to measure the growth of DSEs under salt stress either by growing colonies on liquid media or on solid media. Results showed that the ratio of biomass/diameter did not change between different treatments (data not shown). We therefore used both proxies, colony diameter, and hyphal biomass to record the growth of DSEs. The actively growing hyphae of 2-week old fungal colonies cultivated on potato dextrose agar (PDA) medium (Roth, Karlsruhe, Germany) were cut into plugs (9.5 mm diameter) and transferred to fresh PDA medium enriched with NaCl (Roth, Karlsruhe, Germany) in different concentrations (0, 10, 100, 200, 300, 500 mM NaCl). NaCl concentrations were selected after prior investigation of the responses of the three DSEs to even higher concentrations. Concentrations were selected that can be applied also to plants for our future research on the symbiotic interactions of DSEs with crops under salt stress. For experiments, where mycelial biomass was measured, the fungi were transferred to a sterilized cellophane membrane (Kilner, Liverpool, United Kingdom) covering the medium. Fungi were incubated at 25 ± 1°C in the dark. Radial growth and colony pigmentation were monitored after an incubation of three to 4 weeks, while dry biomass was measured after 2 weeks of growth and subsequent drying at 60°C for 72 h.

### Treatment of DSEs With Melanin Biosynthesis Inhibitors

Inhibition of melanin biosynthesis was carried out by growing *P. macrospinosa*, *Cadophora* sp., and *Leptodontidium* sp. on PDA and Pachlewski agar (Pachlewski et al., 1974) supplemented by the melanin inhibitors kojic acid, sulcotrione, or tricyclazole (Sigma-Aldrich, Munich, Germany) in concentrations of 10, 30, 50 μg/ml for each inhibitor separately. Fungi were incubated at 25 ± 1°C in the dark for different periods (2 and 3 weeks) according to the further analysis.

### Extraction, Purification, and Quantification of Melanin From DSEs

Melanin extraction and purification were carried out from mycelia grown for 2 weeks on PDA according to Zou et al. (2010) with some modifications. Dry mycelium was ground, washed with water for 5 min and treated with 6 M NaOH for adjusting the pH to 12. Then, solutions were sonicated for 24 h, centrifuged at 4,000 rpm for 5 min, and supernatants were acidified with 6 M HCl until reaching pH 2. Mixtures were precipitated for 20 min at 10,000 rpm and pellets were further processed. Purification was conducted by using organic solvents to wash the residues of lipids. Five milliliters of chloroform were added to the pellets, and the solutions were centrifuged for 5 min at 4,000 rpm. The same steps were repeated using ethyl acetate and finally ethanol as solvents. Optical density of purified melanin was measured at 400 nm by a Synergy HT Multi-Mode Microplate Reader (BioTek Instruments, Winooski, VT, United States). The standard curve was established using synthetic melanin (Sigma-Aldrich).

### Quantification of Na and K Contents in *Periconia macrospinosa*

*Periconia macrospinosa* mycelia were harvested 3 weeks after growing under salt stress and with or without 40 μg/mL of the melanin biosynthesis inhibitor tricyclazole. The mycelia were dried at 60°C for 72 h and dry biomass was recorded. Na- and K-containing solutions were obtained by using the following microwave digestion method. Hundred milligram of ground fungal samples were digested with 2.5 mL concentrated HNO_3_ (65%) and 1.5 mL H_2_O_2_ (30%) at 210°C for 45 min in a microwave (MARSXpress 250/50; CEM Corporation, Matthews, NC, United States). Thereafter, deionized water was added to a final volume of 25 ml. Subsequently, Na and K concentrations were determined by emission spectroscopy (ICP-OES, Thermo Fisher Scientific, Dreieich, Germany), using wavelengths of 589 and 592 nm for Na and 766 and 490 nm for K (Gericke and Kurmies, 1952).

### Cloning and Sequencing

All target genes (Table 2) were amplified by PCR with a Primus Thermo cycler (MWG Biotech, Ebersberg, Germany) under the following conditions: 95°C for 4 min, 35 cycles (94°C for 30 s, annealing for 30 s at 60°C, 72°C for 30 s), and a final 10 min extension at 72°C. After confirmation by 1.5% agarose gel electrophoresis, 3 μL PCR products were purified on spin columns according to the manufacturer’s protocol (Roche, Mannheim, Germany), cloned into the pGEM-T Easy vector according to the manufacturer’s protocol (Promega, Mannheim, Germany) and transformed into competent JM109 *Escherichia coli* cells (Promega). Sequencing was conducted by Eurofins MWG Operon (Ebersberg, Germany).

**TABLE 2:** List of genes that were analyzed in the study, JGI accession numbers (http://genome.jgi.doe.gov/Perma1, http://genome.jgi.doe.gov/Cadsp1) and primer sequences.

Genes	Accession No.	Primer sequences	References
*PmSCD*	616001	CGACAAGGTGTTTTCCGAGG GACAATCCATACTACAAGCCGC	The current study
*CadSCD*	585435	GCTTGCCTTACTTCAAATCGGT CCTTCCACTTCCCGTCAATCT	The current study
*PmACT*	610022	TCTCAATCTTCCGCCACCTT CTTGATCTTGGAACCGCTCG	The current study
*CadGPDH*	417926	TTCTGCCAACACGGAACTGT CTTGCCATCTCCAGACTCGG	Yakti et al., 2019

### Analysis of Gene Expression

RNA accumulation analyses of the scytalone dehydratase-encoding gene *SCD* were conducted for *Cadophora* sp. and *P. macrospinosa*. For this purpose, newly grown mycelia of both DSEs were challenged with PDB liquid PDA enriched with 200 and 500 mM or without NaCl as control. Mycelia were harvested after 0, 6, 16, and 24 h of exposure to NaCl and kept at −80°C until further use. Total RNA was extracted from mycelia using an RNA extraction kit (Analytik Jena, Jena, Germany) according to the manufacturer’s instructions. RNA quantity and quality were checked by 1% agarose gel electrophoresis and photometric analysis by NanoDrop1000 Spectrophotometer (Thermo Fisher Scientific). Total RNA was DNase-treated using the RNase-free DNase kit RQ1 (Promega) according to the manufacturer’s instructions. cDNA was synthesized from 2 μg RNA using the M-MLV Reverse Transcriptase kit (Promega) in a 25 μL reaction following the manufacturer’s instructions.

RNA accumulation of target genes (Table 2) was analyzed by Real-time PCR using the 7500 fast real-time PCR system (Applied Biosystems, Foster City, CA, United States). SYBR green (Bioline, Luckenwalde, Germany) was used as fluorescent dye. Three biological replicates and three technical replicates were conducted for each treatment. Each reaction contained a volume of 10 μL including 5 μL SYBR green mix (SYBR green Low-ROX 2× Sensimix, Bioline), 100 nmol/L forward and reverse primers (Table 2) and 10 pg cDNA template. Primers were designed using the genomic information of the two fungi (Knapp et al., 2018). Target genes transcript identification numbers are 616001 (the only *arp1* homolog) and 585435 (the second from three *arp1* homologs) for *P. macrospinosa* and *Cadophora* sp., respectively (Table 2). The amplification program was conducted as follows: 95°C for 10 min, 40 cycles (95°C for 15 s, 60°C for 1 min). A melting curve (95°C for 15 s, 60°C for 1 min, 95°C for 15 s) was recorded at the end of every run to identify reactions where primers generated non-specific PCR products (Ririe et al., 1997). The relative RNA accumulation was expressed as 2^ΔCt^. The relative quantification method was used for calculations according to the formula ΔC_t_ = C_t_ reference gene – C_t_ target gene (Pfaffl, 2001). Reference genes (Table 2) were chosen according to their highest stability among four tested reference genes. The stability of every reference gene expression was assessed by calculating the expression stability (M) value and coefficient of variation (CV) using Biogazelle qBase^+^ version 3.0 (Biogazelle, Zwijnaarde, Belgium) where M < 0.5 and C_V_ < 0.25. Efficiency of qRT-PCR was calculated using LinReg software (University of Amsterdam, Amsterdam, Netherlands). Efficiency values were close to the optimum of 2.0 (Ruijter et al., 2009; Ruijter et al., 2014).

### Statistical Analysis

The statistical analysis of data was performed using Statistica software (version 12, Tulsa, OK, United States). The normal distribution and homogeneity of data were carried out by Kolmogorov–Smirnov and Levene’s tests, respectively. One-way non-parametric Kruskal–Wallis test was carried out for the variables with non-normal distribution. Two-way analysis of variance (ANOVA) was used for data analyses of normally distributed variables. *Post hoc* Tukey HSD and Dunn’s multiple comparison tests were performed at *p* = 0.05. All data are shown as mean values with standard deviations (SD).

## Results

### Response of DSEs to Salt Stress

**FIGURE 1:**
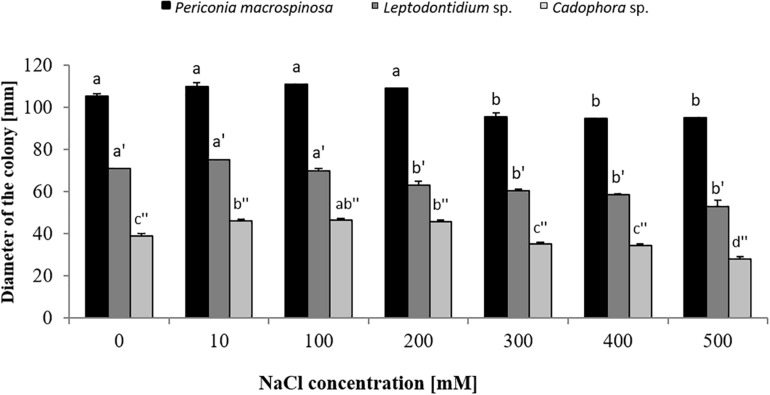
Growth of *Periconia macrospinosa*, *Leptodontidium* sp., and *Cadophora* sp. under salt stress. DSEs were grown on potato dextrose agar (PDA) medium supplemented with different NaCl concentrations for 3 weeks at 25°C. One-way non-parametric Kruskal–Wallis test (*P* = 0.05, *n* = 15; independent for each fungus) was carried out followed by Dunn’s multiple comparison test that showed significant differences between the growth of the DSEs under different salt concentrations and are indicated by different letters. Bars represent standard deviations of the means.

**FIGURE 2:**
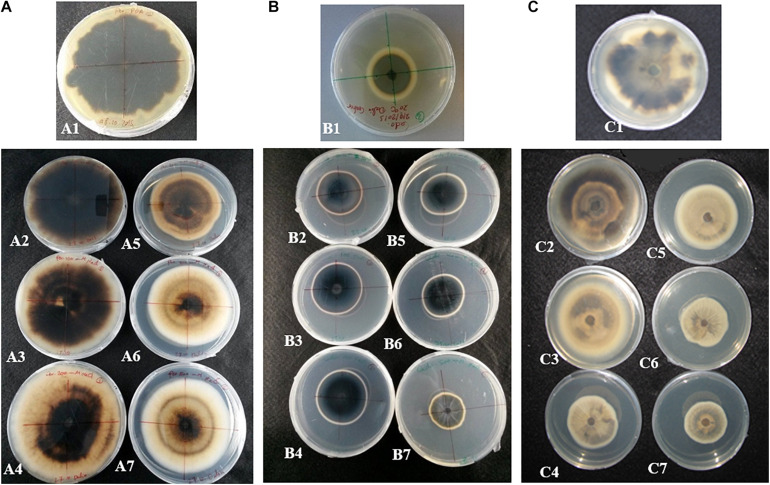
Effect of salt stress on the pigmentation of **(A)** *P. macrospinosa*, **(B)** *Cadophora* sp., and **(C)** *Leptodontidium* sp. Photos show the morphological appearance and coloration of *P. macrospinosa* and *Cadophora* sp. when they were 4 weeks old and cultivated under salt stress. Colonies grown in the presence of 0, 10, 100, 200, 300, 400, and 500 mM NaCl are shown in photos A1–A7, B1–B7, and C1–C7, respectively.

Colony diameters of *P. macrospinosa*, *Cadophora* sp., and *Leptodontidium* sp. were measured after 3 weeks of growth on PDA media enriched with different concentrations of NaCl from 10 up to 500 mM (Figure 1). *Cadophora* sp. grew at a higher rate on media enriched with 100 mM of NaCl than on the control media without salt (Figure 1). The growth of DSEs decreased at 200 mM in *Leptodontidium* sp. and at 300 mM NaCl in *P. macrospinosa* and *Cadophora* sp. Only *Cadophora* sp. was further decreased at 500 mM of NaCl (Figure 1). The melanization of *P. macrospinosa* and *Leptodontidium* sp. was notably inhibited by the increment of salt stress from 10 up to 500 mM NaCl (Figures 2A,C, respectively). The same observation was noted also in case of *Cadophora* sp., but only at 500 mM salt (Figure 2B).

### Scytalone Dehydratase Gene Expression in Hyphae of *Periconia macrospinosa* and *Cadophora* sp. Under Salt Stress

**FIGURE 3:**
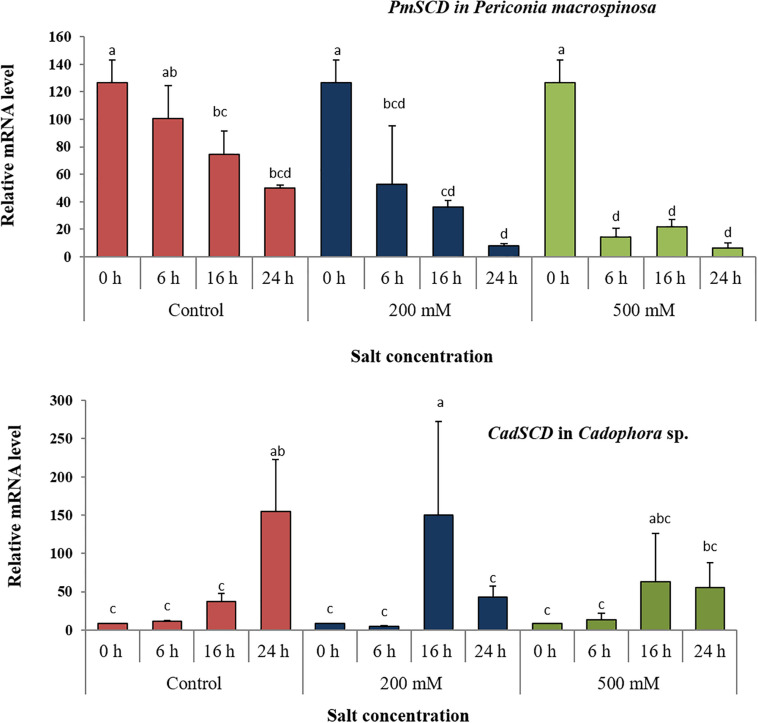
Expression analysis of the scytalone dehydratase encoding gene (*SCD*) under salt stress. Relative RNA accumulation of *PmSCD* and *CadSCD* was analyzed by qRT-PCR using as reference the actin-encoding gene for *P. macrospinosa* and the GAPDH-encoding gene for *Cadophora* sp. at 0, 200, and 500 mM NaCl. Bars represent standard deviations of the means. Two-way ANOVA (*P* = 0.05; *n* = 9) has been applied between the factor salt concentration and time of exposure to salt stress. For each strain, different letters indicate significant differences obtained by Tukey HSD test.

In order to test short-term effects of salt on melanin biosynthesis, the expression of a gene encoding the key enzyme scytalone dehydratase in the DHN pathway was analyzed in *P. macrospinosa* and in *Cadophora* sp. after transfer of the colonies to medium containing 0, 200, and 500 mM NaCl. These concentrations were selected, because they resulted in significant different growth parameters for the two fungi (Figure 1). There are significant interactions according to two-way ANOVA between the factor salt concentration and time of exposure to salt stress. qRT-PCR analysis first revealed that the expression of *PmSCD* was downregulated as a function of time, while *CadSCD* was induced 24 h after transfer to fresh medium (Figure 3). Comparing the values at the same time points at different salt concentrations, *PmSCD* was slightly downregulated at 200 mM and strongly at 500 mM NaCl. The same is true for *CadSCD* at 24 h, but the gene seems to be upregulated at 16 h although SD are rather high. In summary, genes encoding scytalone dehydratase of both DSEs are rather down- than up-regulated by salt treatment.

### Impact of Salt Stress and Tricyclazole on Melanin Production, Fungal Biomass, and Element Accumulation

Before the role of melanin biosynthesis on salt stress tolerance was investigated, the results of Berthelot et al. (2020) obtained for *Leptodontidium* sp. and *Cadophora* sp. were here confirmed for *P. macrospinosa*. After 2 weeks of growth on PDA or Pachlewski media supplemented with the different melanin biosynthesis inhibitors, inhibition of melanin production could be only observed in the presence of tricyclazole (Supplementary Figure 2) suggesting that melanin is synthesized *via* the DHN-pathway in *P. macrospinosa* like in the two other DSEs.

**FIGURE 4:**
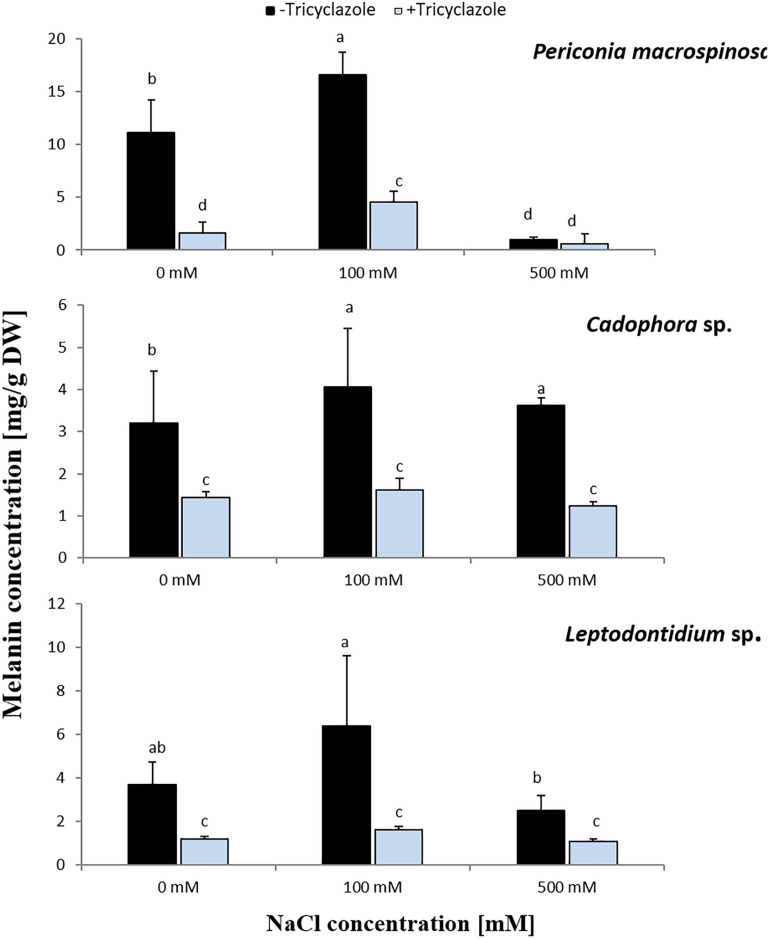
Melanin concentration in hyphae of *P. macrospinosa*, *Cadophora* sp., and *Leptodontidium* sp. Colonies were grown on PDA medium supplemented with different concentrations (0, 100, 500 mM) of NaCl with (+) or without (–) tricyclazole in concentration of 40 μg/mL and incubated at 25°C for 14 days. Bars represent standard deviations of the means. Two-way ANOVA (*P* = 0.05; *n* = 9) has been applied and different letters indicate significant differences between treatments according to Tukey HSD test.

Long-term effects of salt on melanin production was investigated by growing the DSEs on PDA medium for 2 weeks in the presence of medium (100 mM) or high (500 mM) concentration of NaCl with or without tricyclazole. The three fungi revealed significant different growth (Figure 1). Increased melanin production was observed by the addition of 100 mM NaCl in the three fungi and this was significant for *P. macrospinosa* and *Cadophora* sp. (Figure 4). By further increasing the NaCl concentration to 500 mM, melanin production was severely reduced in *P. macrospinosa*, but was still significantly increased in *Cadophora* sp. Melanin production was constantly low in the media supplemented with tricyclazole.

**FIGURE 5:**
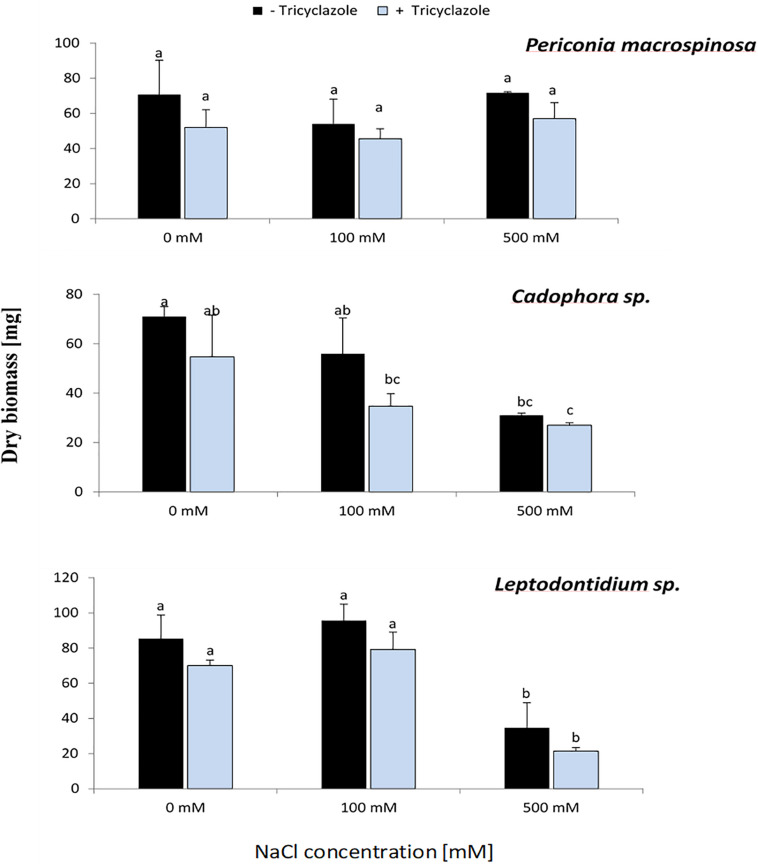
Effect of tricyclazole and salt stress on the growth of *P. macrospinosa*, *Cadophora* sp., and *Leptodontidium* sp. Colonies were grown for 14 days on PDA medium supplemented with different concentrations (0, 100, 500 mM) of NaCl with (+) or without (–) tricyclazole. Bars represent standard deviations of the means. Two-way ANOVA (*P* = 0.05; *n* = 9) has been applied and different letters indicate significant differences between treatments according to Tukey HSD test.

In the same experiment, fungal dry biomass for each DSE was recorded (Figure 5). This showed a similar trend for *Cadophora* sp. and *Leptodontidium* sp. as in the first experiment in which the growth rate was recorded as colony diameter (Figure 1). Significant effects were shown only at 500 mM. Such effect could not be observed for *P. macrospinosa*. More importantly, the inhibition of melanin biosynthesis by tricyclazole had no impact on fungal biomass of all three DSEs, neither in the controls (no salt) nor in the treatments with 100 and 500 mM NaCl.

**FIGURE 6:**
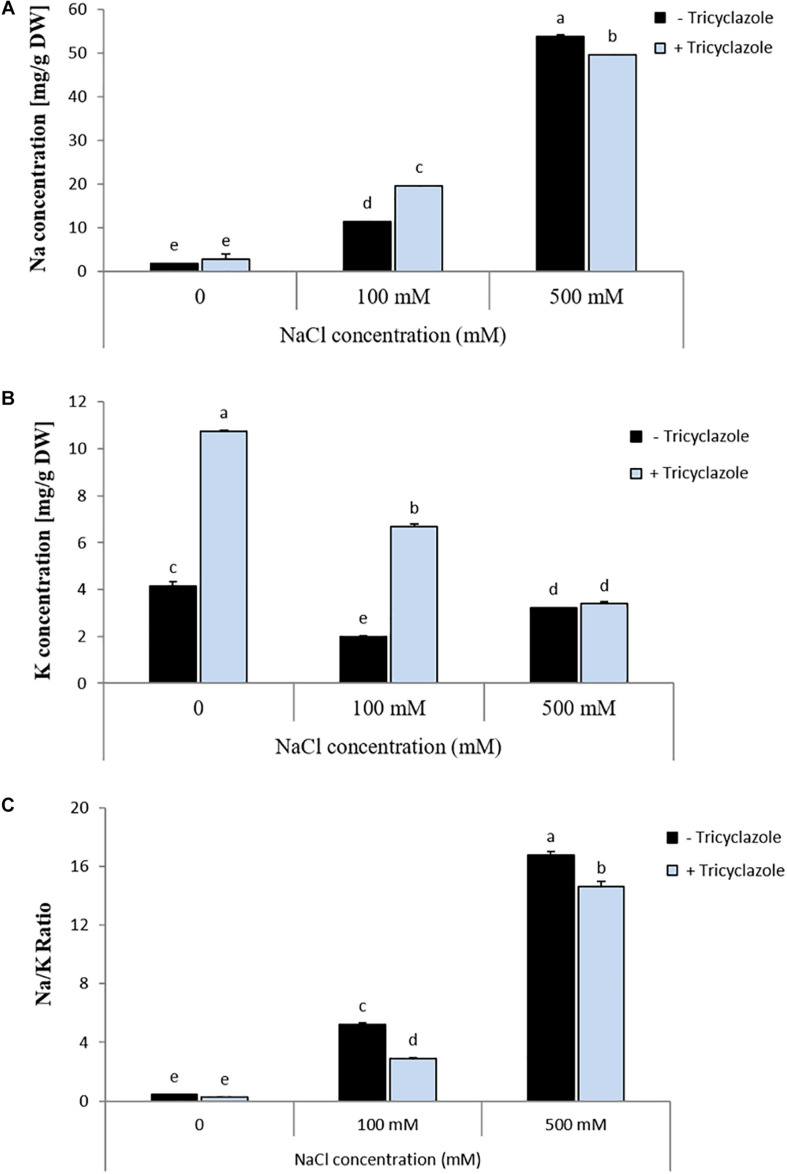
Impact of tricyclazole and salt stress on **(A)** Na concentration, **(B)** K concentration, and **(C)** Na/K ratio in *P. macrospinosa*. The fungus was grown at 25°C on PDA medium supplemented with (light blue bars) or without (black bars) tricyclazole and with salt concentrations of 0, 100, and 500 mM. Mycelia were harvested after 3 weeks of growth. Bars represent standard deviations of the means. Two-way ANOVA (*P* = 0.05; *n* = 3) has been applied and different letters indicate significant differences between treatments according to Tukey HSD test.

Three weeks old *P. macrospinosa* cultures growing on PDA with different salt concentrations and with or without the addition of tricyclazole were used for measuring Na and K accumulation in the mycelia (Figure 6). The Na/K ratio was calculated in the different conditions with salt and tricyclazole treatments. Addition of NaCl to the medium increased the accumulation of Na and decreased the accumulation of K by the hyphae which resulted in higher Na/K ratios in *P. macrospinosa* hyphae compared to hyphae not exposed to salt stress. Treatment with melanin inhibitor led to increased Na and K accumulation at 100 mM salt stress. At 500 mM, the application of tricyclazole decreased Na concentrations and had no effect on K accumulation of *P. macrospinosa* hyphae compared to controls without melanin inhibitor. Under 100 and 500 mM NaCl treatments, Na/K ratio decreased when melanin biosynthesis was inhibited suggesting that melanin did not inhibit NaCl uptake.

### Impact of Salt Stress on Melanin Mutants of *Leptodontidium* sp.

**FIGURE 7:**
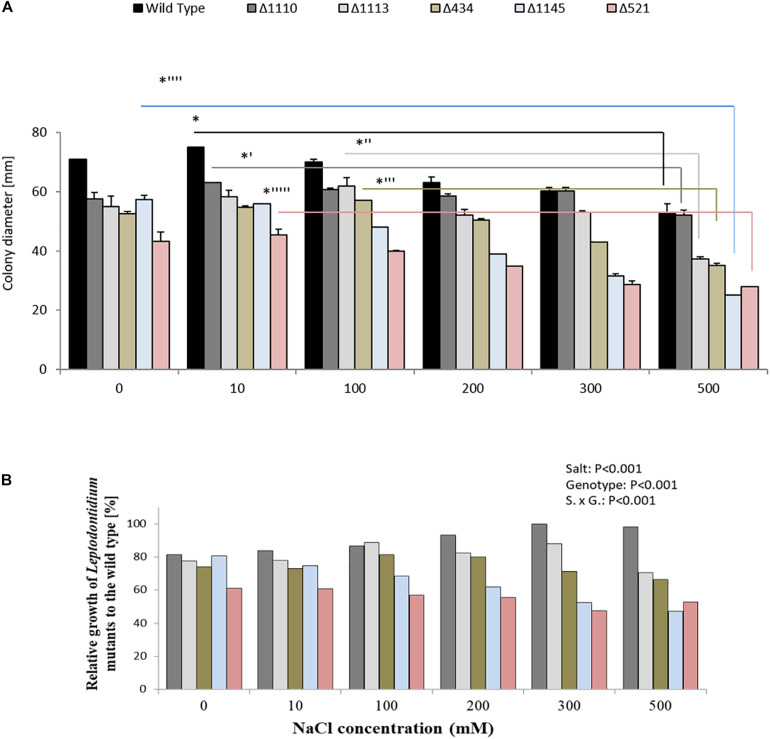
Impact of genotype and salt stress on growth of isogenic albinos and wild-type *Leptodontidium* sp. **(A)** Diameters of fungal mycelia were recorded. Data are shown as means and standard deviations. One-way non-parametric Kruskal–Wallis test (*P* = 0.05; *n* = 15; independent for each fungus) was carried out followed by Dunn’s multiple comparison test that showed significant differences between the growth of *Leptodontidium* sp. genotype under different salt concentrations and are indicated by asterisks. Wild-type in black bars, mutants Δ1110 in dark gray bars, Δ1113 in light gray bars, Δ434 in light green bars, Δ1145 in light blue bars, and Δ521 in rose bars. Bars represent standard deviations of the means. **(B)** Relative growth of albino mutants to the wild-type. Values of the growth of *Leptodontidium* sp. (wild-type) were set to 100%. Bars represent standard deviations of the means. Two-way ANOVA (*P* = 0.05; *n* = 15) shows significant effects of the factors salt and genotype, as well as significant interaction between the factors.

The growth of *Leptodontidium* sp. wild-type and melanin mutants was estimated under salt stress from 0 to 500 mM. In general, the growth of the melanized wild-type and of the non-melanized albino mutants of *Leptodontidium* sp. was affected by salt stress at high concentration (500 mM) of NaCl (Figure 7A). All mutant strains showed smaller colony diameters than the wild-type in the control, but this difference disappeared for some of the mutants and increased for others at higher salt concentrations. For instance, the growth difference between the albino mutant Δ1110 and the wild-type decreased by increment of NaCl concentrations in the medium and statistically disappeared at 200 mM and above. In contrast, the growth difference between the albino mutants Δ1145 and Δ521 increased by high NaCl concentrations (Figure 7B).

## Discussion

Salt stress tolerance is common in various fungal phyla. Among 44 ascomycetous, 8 zygomycetous, and 6 basidiomycetous endophytic fungi isolated from roots of *Pinus thunbergii* in three coastal regions in Korea, 18 species showed salt stress tolerance most of which belonged to the ascomycetous genera *Penicillium* and *Trichoderma* (Min et al., 2014). High salt concentrations up to 1.1 M of NaCl were tolerated by *Trimmatostroma abietis* with optimal growth at 350 mM (Kogej et al., 2006). Mycelial growth, conidia production, and conidium germination of *Verticillium dahliae* was also highest at 256 mM (Regragui and Lahlou, 2005), and *Aspergillus repens* could even grow on medium with 2 M NaCl (Kelavkar et al., 1993). Boumaaza et al. (2015) found that salt stimulates the development of six isolates of *Botrytis cinerea* in concentrations up to 2.6 mM. In contrast, Oomycota appeared to be more sensitive to salt. Pelizza et al. (2007) observed that zoospores of *Leptolegnia chapmanii* showed 100% mortality already at 119.8 mM of NaCl in the culture medium. Preuett et al. (2016) showed that zoospores were not released from sporangia of *Phytophthora ramorum* above 239.5 mM NaCl and sporangia were not produced above 342.2 mM NaCl. Highest salt concentrations can be tolerated by halophilic fungi. Zajc et al. (2014) observed that salt concentrations of 3.4 M of NaCl still stimulate the growth of the halophilic basidiomycetous fungus *Wallemia ichthyophaga.* Here, we investigated the impact of salt stress on three different DSEs, *P. macrospinosa*, *Cadophora* sp., and *Leptodontidium* sp. *Cadophora* sp. grew significantly more in media enriched with low concentrations of NaCl compared to the controls. This could be explained by the fact that the low concentrations of sodium and chloride are in the useful metabolically acceptable range of minerals for the tested fungi. Slight differences could be observed between the three fungi. However, the extent of the reduction, however, was similar for all three DSEs between 300 and 500 mM NaCl. In summary, the three DSEs show moderate salt tolerance, since the growth reduction was not very strong (10–20%) even at 500 mM. Far from being halophilic, they are still well suited objects to study the mechanisms of salt tolerance.

Melanin increased the tolerance of fungi to abiotic stresses such as freezing, heat, UV, drought, metal toxicity, osmotic stress, and hypersalinity (Kogej et al., 2007; Kejzar et al., 2013; Berthelot et al., 2020). It also plays a role in the resistance of *B. cinerea* to mycoparasitic fungi (Musavi et al., 1991). In addition, melanin increases the virulence of pathogenic fungi (Butler and Day, 1998; van Duin et al., 2002; Gessler et al., 2014). This led us to formulate the main hypothesis of the study that melanin plays a role in the moderate salt stress tolerance of DSEs.

We first investigated whether a gene involved in melanin biosynthesis was induced after exposure to salt. In our study, a general down-regulation of *PmSCD* was found after transfer of the fungus to fresh medium (Figure 3). This could be due to the initiation of new hyphal growth. Such developmentally young hyphae of *P. macrospinosa* might show a relatively lower expression of the gene. In *Cadophora* sp., an opposite pattern was observed. *CadSCD* was generally induced at 16 h after the transfer. Whether this gene expression pattern was accompanied by a differentially regulated melanin production depending on the developmental stage of the hyphae in the two fungi was not analyzed, as it was not in the focus of the current study.

In addition to this developmentally regulated pattern, expression of *PmSCD* showed a significant reduction at 500 mM salt exposure, while down-regulation of *CadSCD* was minimal (Figure 3). These expression patterns were consistent with the pigmentation of the colonies (Figure 2) and with the melanin quantification (Figure 4). While *P. macrospinosa* showed lighter pigmentation and reduced melanin concentrations at high salt concentrations, that was not the case with *Cadophora* sp. Although both fungal strains were isolated from the same environment, they belong to distant phylogenetic lineages. This could explain why they showed their particular *SCD* gene expression pattern and differences in melanin accumulation under salt stress. This is also supported by the findings of Knapp et al. (2018). They revealed that the total number of genes associated with DHN melanin synthesis in *Cadophora* sp. and *P. macrospinosa* are 134 and 133, respectively. Analysis of the *P. macrospinosa* genome showed that it possessed much higher numbers of polyketide synthesase (PKS)-related gene homologs than *Cadophora* sp., which carried more genes for scytalone dehydratase (SCD) (homologous to *Arp1, n* = 3), THN reductase (homologous to *Arp2, n* = 79) and *Arp1-2* homologs (*n* = 27) than *P. macrospinosa* (*n* = 10). Beside these differences between the two fungi, the hypothesis that salt stress induces melanin production in the DSEs investigated must be rejected, especially since *P. macrospinosa* showed the highest salt tolerance among the three DSEs, but the lowest melanin concentration after 2 weeks of growth (Figure 4).

Melanin granules have been suggested to support cell wall function in excluding ion influx (Kogej et al., 2007), and melanin granules reduce the permeability of *C. neoformans* cell wall due to pores size reduction (Jacobson and Ikeda, 2005). We tested the hypothesis that melanization of cell walls could increase exclusion and therefore decrease the intracellular content of toxic sodium ions by inhibiting melanin biosynthesis in *P. macrospinosa* (Figure 6). This could only be confirmed at a concentration of 100 mM NaCl at which the Na content in the melanized hyphae of *P. macrospinosa* was lower than the Na content in the non-melanized hyphae. The opposite effect was observed at high salt concentration (500 mM of NaCl). The inhibition did not lead to an increased, but rather a decreased influx of sodium ions indicating that melanin can facilitate the uptake at high salt concentrations in contrast to what has been hypothesized. In case of potassium, exclusion by melanin could be deduced by the application of the inhibitor, if no or 100 mM NaCl were added to the medium, but the effect disappeared at 500 mM NaCl. It seems that the effect of melanin on ion transport across fungal cell walls and the plasma membrane depends on the type of ion. Sequences for three putative Na/K transporters have been detected in the genome of the fungus, but an analysis of the influence of melanin on the putative differential transport capacities for the two ions would have been beyond the scope of the present study.

Potassium and sodium are the most abundant cations in living cells and natural environments. The Na/K ratio becomes toxic above a certain ratio (Rodriguez-Navarro, 2000). One important strategy for alleviating the adverse effects of salinity on the cells, the ionic homeostasis, is the maintenance of the Na/K balance. In the present study, *P. macrospinosa* showed a significantly lower Na/K ratio when exposed to tricyclazole compared to the controls at 100 and 500 mM salt in the medium. This suggests that melanin does not play a positive, but rather a negative role in ionic homeostasis in the hyphae of *P. macrospinosa* and this is probably due to the differential effect on the transport of Na and K.

If melanin had a protective effect against salt stress, the inhibition of melanin biosynthesis should lead to a decreased salt stress tolerance. As expected, the application of the inhibitor tricyclazole led to a reduced melanin concentration in the three DSEs with the exception of *P. macrospinosa* at 500 mM NaCl, nevertheless, in this case the melanin concentration was already low without inhibitor application (Figure 5). The growth of the three DSEs was slightly reduced in the presence of the inhibitor, and this could already be observed without the presence of salt. Tricyclazole could not only inhibit the melanin production, but also reduce sporulation, spore size, and number of septa in conidia, as described by Kawamura et al. (1999) and Chattopadhyay et al. (2013). This could explain the slight inhibition of the growth of the three DSEs in the presence of tricyclazole, which was however not significant. The hypothesis that melanin inhibition decreases salt tolerance must therefore be rejected.

Biochemical approaches in the study of melanin synthesis are often criticized, because inhibitors such as tricyclazole could have additional unexpected effects that distort the results (Bashyal et al., 2010; Chattopadhyay et al., 2013). In order to obtain independent evidence for the finding, a genetic approach was chosen. If melanin plays a role in salt tolerance, it would be expected that all albino mutants that are impaired in melanin biosynthesis will be more sensitive to salt stress compared to the wild-type. In fact, this was not the case (Figure 7). No mutant strains showed significant growth alteration even with 500 mM NaCl in the medium. This confirmed the result of the inhibitor experiments and supported the decision to reject the hypothesis that melanin plays a role in salt tolerance.

## Conclusion

In summary, the hypothesis that melanin plays a role in the sodium chloride salt tolerance of the three different DSEs under the conditions tested could be rejected. *Leptodontidium* sp. albino mutants impeded in melanin production showed no lower salt tolerance than the corresponding wild-type. Melanin biosynthesis in *P. macrospinosa* and *Cadophora* sp. was not induced by salt treatment. In addition, melanin inhibition does not lead to an increased sodium influx into *P. macrospinosa* hyphae and to a reduced salt tolerance in all three DSEs. However, it cannot be ruled out that melanin is important for tolerance to salt concentrations that are much higher than in this study, which can be found in certain ecosystems such as salt marshes. Nor can it be ruled out, that melanin plays a role in conferring salt stress tolerance to the plant if DSEs colonize the roots. The role of melanin in the interaction with the plant was not the subject of the current study, but certainly deserves further investigation. Melanin could also play a role in tolerances to other abiotic stresses. Protection against heavy metals does not appear to depend on melanin. Berthelot et al. (2020) showed that the uptake of Zn and Cd increased neither in albino mutants nor in colonies treated with tricyclazole. The current experiments and those of Berthelot et al. (2020) showed a general inhibition of growth. Whether this is actually due to the lack of melanin or due to side effects of the inhibitor and insertions in other parts of the genome remains to be proven.

## Data Availability Statement

The datasets presented in this study can be found in online repositories. The names of the repository/repositories and accession number(s) can be found in the article/Supplementary Material.

## Author Contributions

DG and PF designed the research, interpreted the data, and wrote the manuscript. DG performed the research and analyzed the data. GK, DB, and CB provided the endophytes under investigations and co-supervised the experimental work. All authors contributed to revising the manuscript and approved the submitted version.

## Conflict of Interest

The authors declare that the research was conducted in the absence of any commercial or financial relationships that could be construed as a potential conflict of interest.
