# Supplementary material for: Salt Stress Tolerance of Dark Septate Endophytes Is Independent of Melanin Accumulation
Source: Front Microbiol. 2020 Dec 10;11:562931. doi: 10.3389/fmicb.2020.562931 (PMC7758464; doi:10.3389/fmicb.2020.562931)
Supplement: Supplementary file 1 [file Data_Sheet_1.docx]

Supplementary Material


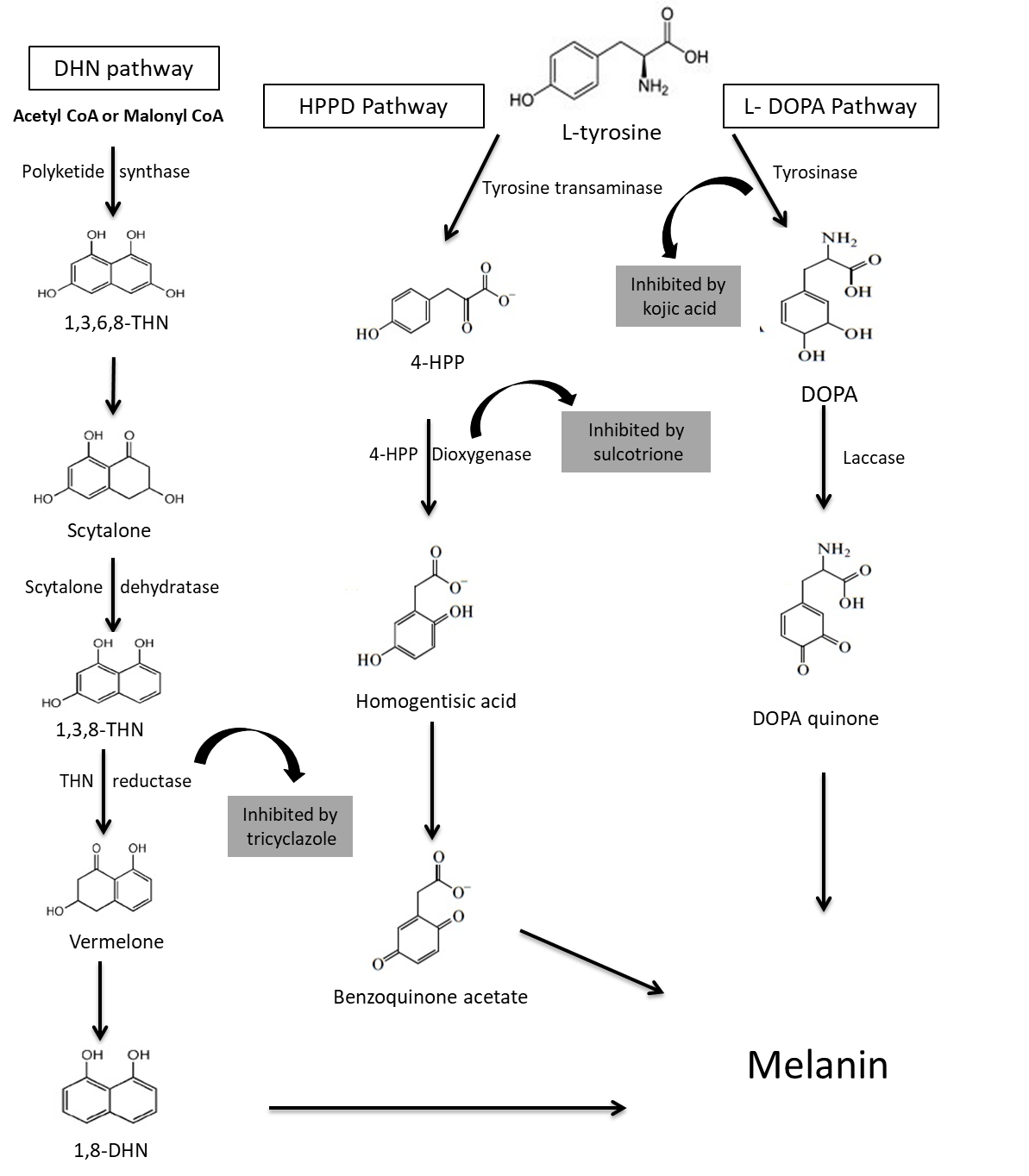


**Figure S1: Illustration of the three possible melanin biosynthesis pathways in fungi and the melanin inhibitor for each pathway (Wheeler and Klich 1995; Bell and Wheeler 1986; Butler and Day 1998; Langfelder et al. 2003; Cabanes et al. 1994; Carreira et al. 2001; Coon et al. 1994; Secor 1994).**

1,3,6,8-THN: 1,3,6,8 tetrahydroxynaphthalene, 1,3,8-THN: 1,3,8-trihydroxynaphthalene, 1,8-DHN: 1,8-dihyroxynaphthalene and DOPA: 3,4 dihydroxyphenylalanine.


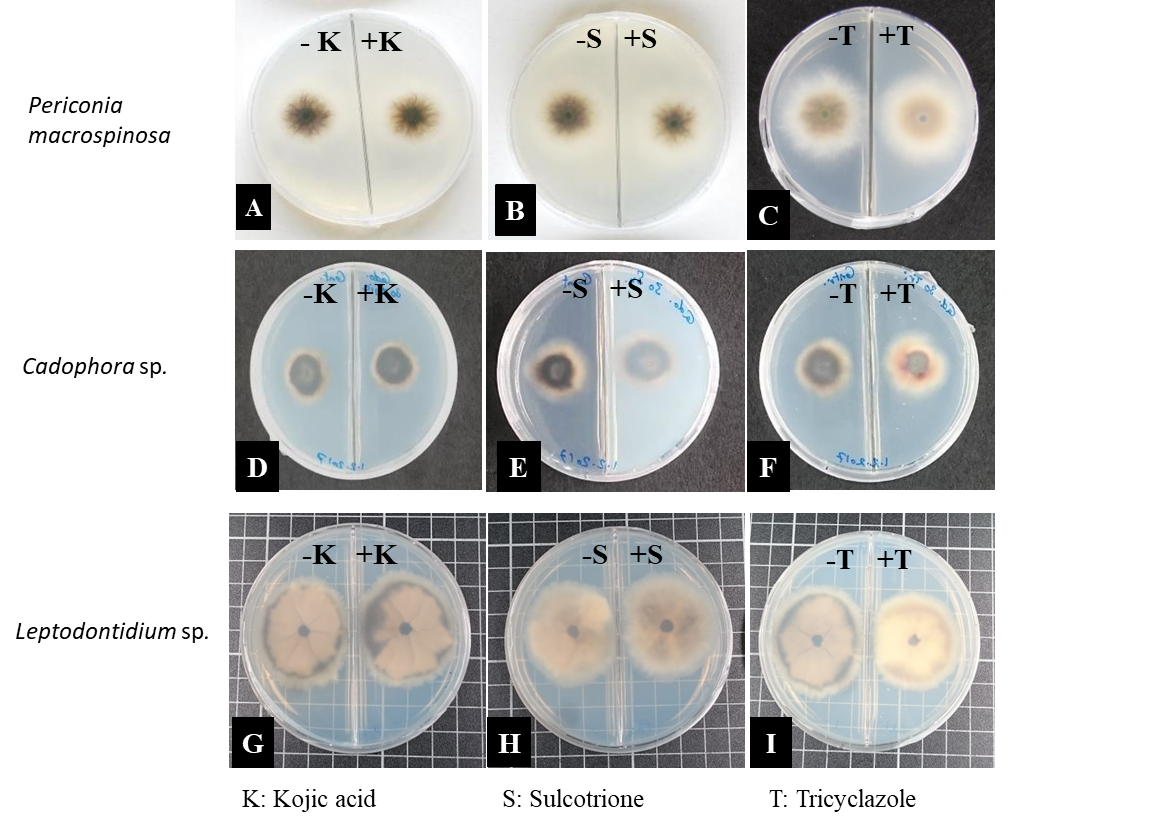


**Figure S2: Impact of three melanin biosynthesis inhibitors on the pigmentation of *Periconia macrospinosa, Cadophora* sp. and *Leptodontidium* sp.** DSEs were grown on Pachlewski media enriched with either kojic acid (K), sulcotrione (S) or tricyclazole (T). Petri dishes were incubated at 25^o^C for 14 days. Melanin pigmentation were not inhibited in the presence of kojic acid and sulcotrione in *P. macrospinosa* (A and B), *Cadophora* sp. (D and E) and *Leptodontidium* sp. (G and H), while the melanin pigment was reduced by the addition of tricyclazole visible in *P. macrospinosa* (C), *Cadophora* sp. (F) and *Leptodontidium* sp. (I). The experiment has been repeated 3 times with 5 repetitions per each treatment obtaining similar results.

**References:**

Bell AA, Wheeler MH (1986) Biosynthesis and functions of fungal melanins. Annual Review of Phytopathology 24:411-451. doi:10.1146/annurev.py.24.090186.002211

Butler MJ, Day AW (1998) Fungal melanins: a review. Canadian Journal of Microbiology 44 (12):1115-1136. doi:10.1139/cjm-44-12-1115

Cabanes J, Chazarra S, Garciacarmona F (1994) Kojic acid, a cosmitic skin whitening agent, is a slow-binding inhibitor of catecholase activity of tyrosinase. Journal of Pharmacy and Pharmacology 46 (12):982-985. doi:10.1111/j.2042-7158.1994.tb03253.x

Carreira A, Ferreira LM, Loureiro V (2001) Brown pigments produced by *Yarrowia lipolytica* result from extracellular accumulation of homogentisic acid. Applied and Environmental Microbiology 67 (8):3463-3468. doi:10.1128/aem.67.8.3463-3468.2001

Coon SL, Kotob S, Jarvis BB, Wang SJ, Fuqua WC, Weiner RM (1994) Homogentisic acid is the product of MelA, which mediates melanogenesis in the marine bacterium *Shewanella colwelliana* D. Applied and Environmental Microbiology 60 (8):3006-3010

Langfelder K, Streibel M, Jahn B, Haase G, Brakhage AA (2003) Biosynthesis of fungal melanins and their importance for human pathogenic fungi. Fungal Genetics and Biology 38 (2):143-158. doi:10.1016/s1087-1845(02)00526-1

Secor J (1994) Inhibition of barnyardgrass 4-hydroxyphenylpyruvate dioxygenase by sulcotrione. Plant Physiology 106 (4):1429-1433. doi:10.1104/pp.106.4.1429

Wheeler MH, Klich MA (1995) The effects of tricyclazole, pyroquilon, phthalide, and related fungicides on the production of conidial wall pigments by *Penicillium* and *Aspergillus* species. Pesticide Biochemistry and Physiology 52 (2):125-136. doi:10.1006/pest.1995.1037
